# Systemic acquired resistance in soybean is regulated by two proteins, Orthologous to Arabidopsis NPR1

**DOI:** 10.1186/1471-2229-9-105

**Published:** 2009-08-05

**Authors:** Devinder Sandhu, I Made Tasma, Ryan Frasch, Madan K Bhattacharyya

**Affiliations:** 1 Department of Agronomy, Iowa State University, Ames, IA 50011, USA; 2 Department of Biology, University of Wisconsin-Stevens Point, Stevens Point, WI 54481, USA; 3 Current address: The Indonesian Center for Agricultural Biotechnology and Genetic Resources Research and Development, Jl. Tentara Pelajar 3A Bogor 16111, Indonesia

## Abstract

**Background:**

Systemic acquired resistance (SAR) is induced in non-inoculated leaves following infection with certain pathogenic strains. SAR is effective against many pathogens. Salicylic acid (SA) is a signaling molecule of the SAR pathway. The development of SAR is associated with the induction of pathogenesis related (*PR*) genes. Arabidopsis *non-expressor *of *PR1 *(*NPR1*) is a regulatory gene of the SA signal pathway [1-3]. SAR in soybean was first reported following infection with *Colletotrichum trancatum *that causes anthracnose disease. We investigated if SAR in soybean is regulated by a pathway, similar to the one characterized in Arabidopsis.

**Results:**

Pathogenesis-related gene *GmPR1 *is induced following treatment of soybean plants with the SAR inducer, 2,6-dichloroisonicotinic acid (INA) or infection with the oomycete pathogen, *Phytophthora sojae*. In *P. sojae*-infected plants, SAR was induced against the bacterial pathogen, *Pseudomonas syringae *pv. glycinea. Soybean *GmNPR1-1 *and *GmNPR1-2 *genes showed high identities to Arabidopsis *NPR1*. They showed similar expression patterns among the organs, studied in this investigation. *GmNPR1-1 *and *GmNPR1-2 *are the only soybean homologues of *NPR1*and are located in homoeologous regions. In *GmNPR1-1 *and *GmNPR1-2 *transformed Arabidopsis *npr1-1 *mutant plants, SAR markers: (i) *PR-1 *was induced following INA treatment and (ii) *BGL2 *following infection with *Pseudomonas syringae *pv. tomato (*Pst*), and SAR was induced following *Pst *infection. Of the five cysteine residues, Cys^82^, Cys^150^, Cys^155^, Cys^160^, and Cys^216 ^involved in oligomer-monomer transition in NPR1, Cys^216^ in GmNPR1-1 and GmNPR1-2 proteins was substituted to Ser and Leu, respectively.

**Conclusion:**

Complementation analyses in Arabidopsis *npr1-1 *mutants revealed that homoeologous *GmNPR1-1 *and *GmNPR1-2 *genes are orthologous to Arabidopsis *NPR1*. Therefore, SAR pathway in soybean is most likely regulated by *GmNPR1 *genes. Substitution of Cys^216 ^residue, essential for oligomer-monomer transition of Arabidopsis NPR1, with Ser and Leu residues in GmNPR1-1 and GmNPR1-2, respectively, suggested that there may be differences between the regulatory mechanisms of GmNPR1 and Arabidopsis NPR proteins.

## Background

Plants use a series of physical, preformed chemical and inducible defense mechanisms to protect themselves from pathogen attack. One of the most common inducible defense mechanisms is systemic acquired resistance (SAR). SAR can be triggered by infection with certain pathogenic strains. The induced resistance is typically effective against a wide range of pathogens including those taxonomically unrelated to the SAR inducing organism [4].

Salicylic acid (SA) is a signaling molecule of the SAR pathway [2,5]. Exogenous application of SA increases the resistance of tobacco plants to tobacco mosaic virus (TMV) [6]. SAR can be induced effectively by exogenous applications of either SA or synthetic functional analogs of SA, 2,6-dichloroisonicotinic acid (INA) and benzo (1,2,3) thiadiazole-7-carbo-thioic acid S-methyl ester (BTH) [5,7]. In addition to signaling SAR, SA regulates both basal and *R*-gene mediated local disease resistance mechanisms [8].

The development of SAR is associated with the induction of pathogenesis related (*PR*) gene expression [6]. Increases in the endogenous SA levels in the pathogen-inoculated plants coincide with the increased levels of the *PR *gene expression and enhanced disease resistance [9,10]. Transgenic plants expressing the bacterial salicylate hydroxylase (*nahG*) gene cannot accumulate SA and fail to express SAR development [2,11]. The *PR *genes, known as the SAR markers, have been identified from several plant species including tobacco and Arabidopsis [4]. A soybean *PR1 *homolog, *GmPR1 *is induced by both SA treatment and infection of soybean leaves with *soybean mosaic virus *(SMV) [12].

***n****on-expressor of ****PR1 ***(*NPR1*) is a regulatory gene of the SA signal pathway [1-3]. *NPR1 *is also known as ***n****on-inducible ****im****munity 1 *(*NIM1*) [3] or *salicylic acid insensitive 1 *(*SAI1*)[13]. The *NPR1 *gene encodes a protein containing a bipartite nuclear localization sequence and two protein-protein interactive domains, a multiple ankyrin repeat domain and a BTB/POZ domain [14-16]. Both motifs mediate the interactions of NPR1/NIM1 protein with other proteins. NPR1 is an oligomeric, cytosolic protein. Either following pathogenic infection or in response to SA treatment, NPR1 oligomer becomes monomer and moves into the nucleus to activate transcription of pathogenesis-related (*PR*) genes [17]. The NPR1 protein is also homologous to the Iκ-B and the cactus regulatory proteins found in vertebrates and flies, respectively [3,18]. Both genes are involved in pathways controlling innate immunity in animals. The *npr1 *mutants with mutations in *NPR1 *are sensitive to SA toxicity. In the *npr1 *mutant plants, induction of *PR *genes and pathogen resistance by SA are abolished. In spite of their ability to accumulate SA, mutant plants are unable to induce SAR indicating that NPR1 is required for the SAR signal transduction pathway [14].

SAR inducers have been used in various field studies on several crop plants to reduce disease incidence [19]. In all of these studies, SAR inducers led to reduced disease symptom development. Overexpression of Arabidopsis *NPR1 *or its orthologues in transgenic plants has been shown to induce broad-spectrum resistance. For example, overexpression of *NPR1 *led to development of constitutive enhanced resistance against the bacterial pathogen *Pseudomonas syringae *and the oomycete pathogen *Hyaloperonospora parasitica *in Arabidopsis [20]. Overexpression of *NPR1 *and the rice homolog of *NPR1, NH1 *resulted in enhanced resistance against the blast pathogen, *Xanthomonas oryzae *pv. *oryzae *in transgenic rice [21,22]. In tomato, overexpression of the Arabidopsis *NPR1 *gene resulted in an enhanced level of resistance to bacterial and *Fusarium *wilts and a moderate level of resistance against gray leaf spot and bacterial spot diseases [23]. Similarly, wheat plants transformed with Arabidopsis *NPR1 *resulted in enhanced resistance against *Fusarium graminearum *that causes *Fusarium *head blight in wheat and barley [24]. These studies suggest that manipulated expression of *NPR1 *or its orthologues can create broad-spectrum resistance in crop plants, and therefore, could be a suitable strategy in improving crop plants for disease resistance [25].

In the United States, soybean suffers annual yield losses valued at more than 2.6 billion dollars from various pathogenic diseases [26]. SAR in soybean was first reported following infection with *Colletotrichum trancatum *that causes anthracnose disease [27]. A significant reduction in lesion sizes following *C. trancatum *infection was noted in epicotyls, when cotyledons were pre-injected with *C. trancatum *and *C. lagenarium *spore suspensions [27]. We investigated if SAR in soybean is regulated by a pathway, similar to the one characterized in Arabidopsis. We have shown that there are two orthologous *NPR1 *copies in soybean. Non conservation of the Arabidopsis Cys^216 ^residue in GmNPR1s suggests that either conserved Cys^82^, Cys^150^, Cys^155^, Cys^160 ^residues are sufficient for GmNPR1s' monomerization or some other soybean cysteine residue(s) complements the Arabidopsis Cys^216 ^function.

## Results

### INA induces the *PR-1 *gene expression in soybean

Earlier a soybean *PR1 *homolog, *GmPR1 *was shown to be induced by both SA treatment and infection of soybean leaves with SMV [12]. It has not been shown if SA can systemically trigger the expression of *GmPR1*. We determined if *GmPR1 *is systemically induced in leaves following feeding of soybean roots with INA, a functional analog of SA.

**Figure 1:**
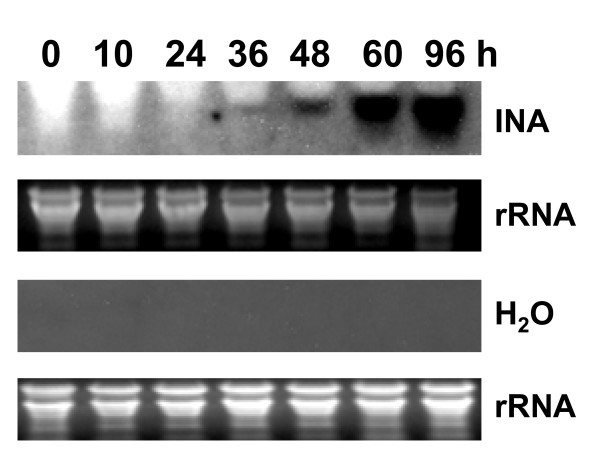
**Induction of the soybean *PR-1 *(*GmPR1*) gene by INA**. Transcripts levels in three-week old soybean seedlings are shown at various hours following feeding with either 0.5 mM INA or water through the roots. Two young trifoliate leaves per plant were harvested at the indicated time points for RNA isolation. For 0 h treatment, the leaves were harvested just before INA treatments. RNA gel blot analysis was performed using the *GmPR1 *gene as the probe. h, hour.

We used INA, a functional analog of SA, to induce *GmPR1*. We investigated the time course accumulation of *GmPR1 *transcripts in response to INA treatment and the data are presented in Figure 1. Northern blot analysis of 3-week old INA treated soybean seedlings showed that *GmPR1 *transcripts were detected as early as 36 h following INA treatment; and thereafter, *GmPR1 *expression levels continued to increase during rest of the time course. These results confirmed earlier observation of SA-mediated *GmPR1 *expression in soybean leaves [12].

### Induction of *PR-1 *gene expression in systemic soybean leaves following *Phytophthora sojae *infection

**Figure 2:**
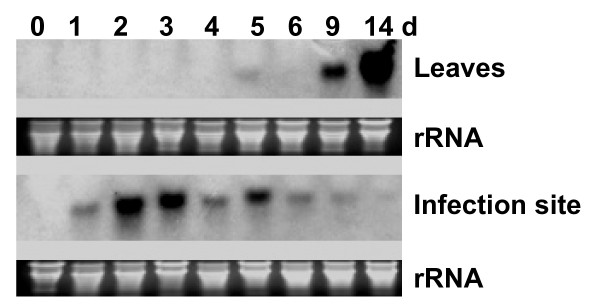
**Induction of *GmPR1 *following infection of hypocotyls with *Phytophthora sojae***. Hypocotyls of 8-day old Williams 82 seedlings were inoculated with *P. sojae *race 4 (avirulent strain). The unifoliate and trifoliate leaves and infected hypocotyl tissues were collected for RNA preparations. Northern analysis was performed using *GmPR1 *as the probe. For 0 day treatment, the leaf and stem tissues were harvested just before inoculation. d, day.

Although it was demonstrated earlier that *GmPR1*, a SAR marker, was induced in response to SMV infection, it has not been shown if *GmPR1 *is systemically induced in non-inoculated systemic leaves [12]. For SAR, induction of *GmPR1 *gene, a SAR marker, is needed in non-inoculated systemic tissue to provide resistance against secondary infection. To determine if pathogenic infection can also lead to *GmPR1 *expression in systemic tissues, hypocotyls of young soybean seedlings were inoculated with an avirulent *P. sojae *race and *GmPR1 *expression was monitored at the site of infection and in non-inoculated systemic leaves. Induction of *GmPR1 *at infection sites was observed as early as on day 1 with a peak on day 2 post inoculation; and thereafter, induction continued until day 9 following inoculation (Figure 2). In the systemic leaves, induction of *GmPR1 *was clearly observed by day 9 following inoculation (Figure 2). No systemic induction of *GmPR1 *was observed when only agar medium with no *P. sojae *mycelia was used to inoculate the wounded hypocotyls (data not shown). These results suggested that SAR pathway is active in soybean.

### Induction of SAR following *Phytophthora sojae *infection

**Figure 3:**
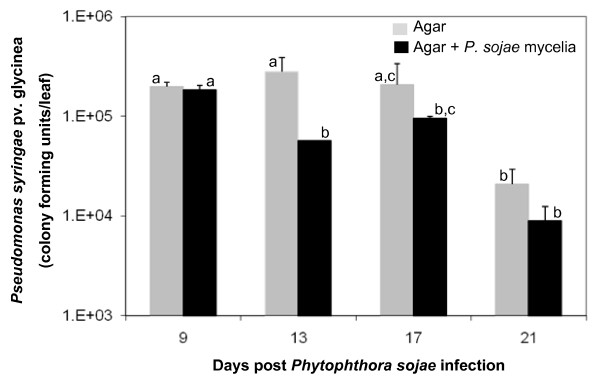
**SAR induction following *Phytophthora sojae *(avirulent) infection in soybean**. Colony forming units (cfu) of *P. syringae *pv. glycinea *(Psg) *per leaf in the samples inoculated with *Psg *9, 13, 17 and 21 days following exposure of wounded hypocotyls to agar pieces containing either no *P. sojae *mycelia (solid gray) or *P. sojae *mycelia (solid black) are shown. Ten microliter droplets of either bacterial cell suspensions (10^7 ^cells/ml) or 10 mM magnesium chloride were used to inoculate the youngest trifoliate. The study was conducted with three biological replications. Bars without a common letter on the top are statistically different (Fisher's LSD test, P = 0.05). Standard errors are represented by *error bars*.

Field studies suggested that SAR was induced following infection of soybean with certain pathogens [27]. Based on the results presented in Figure 2, we designed an experiment to investigate the extent of SAR induction in soybean. Wounded hypocotyls of 7-day old seedlings were inoculated with avirulent strain of *P. sojae *and subsequently at 9, 13, 17 and 21 days after the inoculation leaves were infected with a virulent bacterial pathogen, *P. syringae *pv. glycinea (*Psg*). Four days following *Psg *inoculation colony forming units (cfu) of the pathogen in infected leaves were determined. Bacterial counts were comparable to that in agar control when leaves were inoculated with the bacterium nine days following *P. sojae*-infection (Figure 3). Bacterial counts were, 4.9, 2.2 and 2.3 times lower than the agar-controls when leaves were inoculated with *Psg *13, 17 and 21 days following *P. sojae*-infection. However, only at 13 day the difference was statistically significant (Figure 3). These observations suggested that SAR was induced in non *P. sojae *inoculated soybean leaves following hypersensitive response [28] caused by an avirulent *P. sojae *race.

### Soybean genome contains two copies of *NPR1*-like sequences

**Figure 4:**
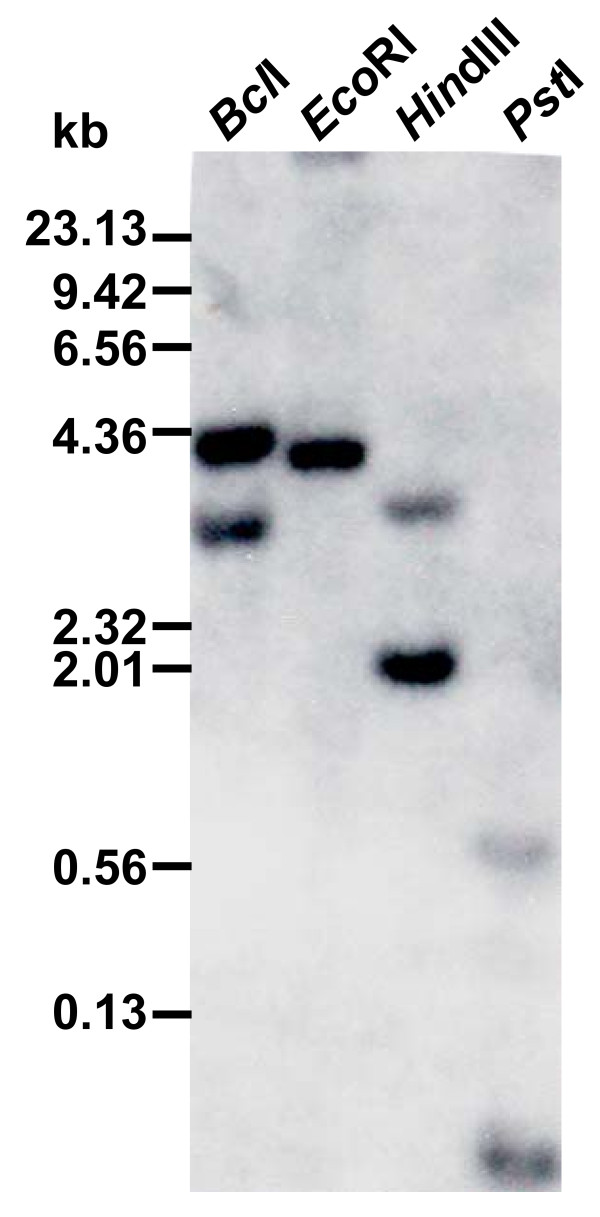
**Genomic organization of *GmNPR1***. Genomic DNA prepared from leaves of the cultivar Williams 82 and digested with four restriction enzymes suggested that there are two copies of *GmNPR1 *in the soybean genome.

**Figure 5:**
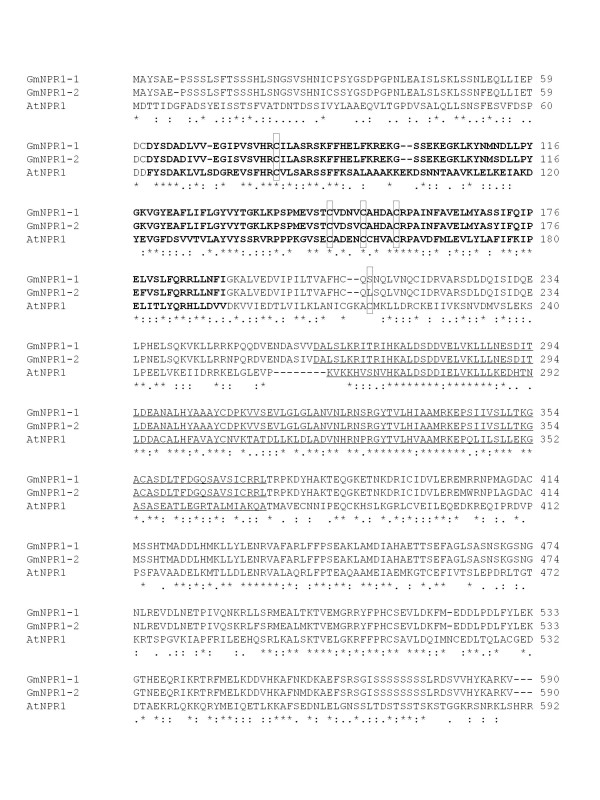
**Comparison of GmNPR1-1 and GmNPR1-2 sequences with that of Arabidopsis NPR1**. Broad-complex Tramtrack Bric-a-brac/Poxvirus and Zinc finger domain (BOB/POZ) is represented by bold letters and Ankyrin repeat domain (ANK) is underlined. Five Arabidopsis cysteine residues (Cys^82^, Cys^150^, Cys^156^, Cys^160 ^and Cys^216^) regulating NPR1 functions are marked with rectangular boxes. "*" represents identical residues; ":" means conserved substitutions between similar residues; "." indicates the semi-conserved substitutions between similar residues.

As a first step towards investigating the molecular components of the SAR pathway in soybean, we determined if the soybean genome contains the orthologue of SAR regulatory gene, Arabidopsis *NPR1*. A 1.7 kb fragment of a candidate soybean *NPR1 *homolog was PCR-amplified from the soybean genomic DNA and named *GmNPR1*. DNA gel blot analysis using the *GmNPR1 *probe revealed that there are two copies of *NPR1*-like sequences in the soybean genome (Figure 4). Screening of a soybean bacterial artificial chromosome (BAC) library [29] for *GmNPR1*-like sequences resulted in identification of 18 BAC clones. DNA fingerprints of these clones for six restriction endonucleases allowed us to group these clones into two classes, Class I and Class II. None of the BAC clones contained both classes of *NPR1*-like sequences suggesting that they are unlikely tandem genes. Screening of a soybean cDNA library prepared from etiolated hypocotyls with *GmNPR1 *resulted in identification of 19 putative clones. These clones were also grouped into two classes based on their restriction patterns. One near full-length cDNA clone for each *GmNPR1*-like sequence was sequenced. We named these two *NPR1*-like sequences, *GmNPR1-1 *(Accession No. FJ418595) and *GmNPR1-2 *(Accession No. FJ418597). *GmNPR1-1 *and *GmNPR1-2 *cDNAs share 96% amino acid identity. Both GmNPR1-1 and GmNPR1-2 shared 40% amino acid identity with Arabidopsis NPR1 (AAC49611) (Figure 5). The cDNA sequences were identical with their corresponding genomic sequences obtained from plasmids p143K5Xb1-2.1 (*GmNPR1-1*) (Accession No. FJ418594) and p101F23E1-2 (*GmNPR1-2*) (Accession No. FJ418596). Data obtained from DNA blot analysis and characterization of BAC and cDNA clones strongly indicated that the diploidized tetraploid soybean contained two *NPR1*-like sequences. In order to confirm this conclusion, we conducted nucleotide sequence comparison of the *GmNPR1 *genes with the soybean genome sequence . *GmNPR1 *genes were identified in two scaffolds (scaffolds_159 and _213) of the soybean genome sequence. *GmNPR1-1 *is located in Scaffold_159 and *GmNPR1-2 *in Scaffold_213. Flanking regions of the two genes were compared for possible microcolinearity. High conservation of gene sequences between the two genomic regions suggested that the two *GmNPR1 *genes are homoeologous and were evolved during the polyploidization event (Additional File 1).

We investigated if there were any additional *GmNPR1*-like sequences in the soybean genome. We conducted search for similar soybean EST sequences using tblastx program (). This led to identification of a GmNPR1-1-like sequence (BE801977.1) with 58% amino acid identity to GmNPR1-1. Duplicated copies of this sequence, *GmNPR1-1*-*like*-*1 *and *GmNPR1-1-like-2*, were identified from Scaffolds_15 and _90 of the soybean genome sequence . These two genes are located in homoeologous regions suggesting that they were also duplicated during polyploidization event (Additional File 2). No significant nucleic acid identity of these two *GmNPR1-1*-like sequences to either of the *GmNPR1 *genes was observed. Proteins encoded by these two homoeologous genes are truncated and do not contain more than 110 residues of the N-terminal core BTB/POZ domain required for SA-mediated activation of *PR1 *(Additional File 3; [30]). Thus, most unlikely they are involved in SAR pathway.

### *GmNPR1 *genes are constitutively expressed in soybean

**Figure 6:**
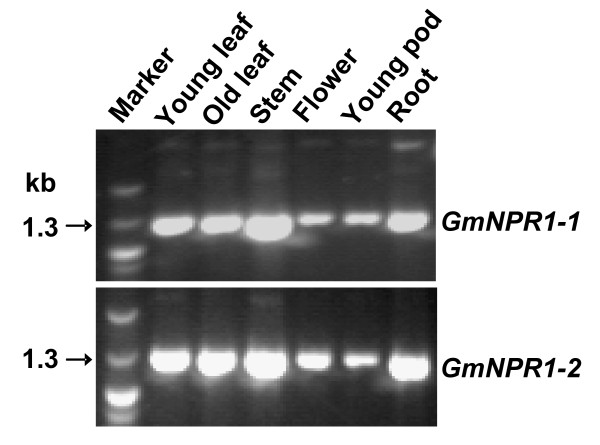
**Constitutive expression of *GmNPR1 *genes among soybean organs**. The arrows indicate RT-PCR products of the *GmNPR1 *genes. Corresponding genomic DNA of the targets for RT-PCR carry introns; and, therefore, amplified products from genomic DNA are much bigger than those from reverse transcribed products. Same reverse transcribed cDNA templates were used for studying transcript profiles of both genes. Therefore, patterns of expression of both *GmNPR1 *genes are comparable and constitutive.

To study the expression patterns of *GmNPR1 *genes, RT-PCR analyses were conducted using gene-specific primers on young and old leaves, stems, flowers, young pods, and roots. Presence of an intron distinguished the PCR products of contaminating genomic DNA from that of the reverse transcribed (RT) cDNA templates for *GmNPR1 *genes. *GmNPR1-1 *and *GmNPR1-2 *were constitutively expressed in all soybean organs investigated (Figure 6). RT-PCR analyses of both genes were conducted using the same RT-templates. Therefore, patterns of steady state transcript levels of both genes in various organs were comparable (Figure 6).

### *GmNPR1 *genes complemented the Arabidopsis *npr1-1 *mutant

**Figure 7:**
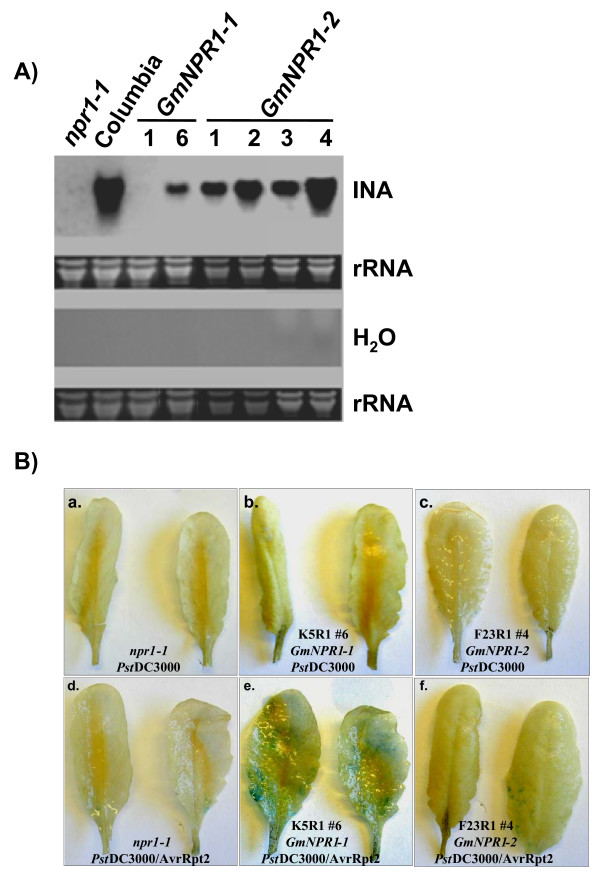
**Induction of the pathogenesis-related genes by INA or infection in Arabidopsis *npr1-1 *mutant carrying *GmNPR1 *genes**. A), Induction of *PR-1 *gene by INA. RNA gel blot analysis was performed using the Arabidopsis *PR-1 *gene as the probe. *GmNPR1-1*; two independent transformants; *GmNPR1-2*, four independent transformants. Note that *PR-1 *is induced in *GmNPR1-1 *and *GmNPR1-2 *complemented *npr1-1 *plants. B), Induction of beta glucanase 2 (*BGL2*) following infection. The leaves of the Arabidopsis *npr1-1 *mutant carrying the *BGL2-GUS *fusion gene with the *BGL2 *promoter transformed with no *GmNPR1 *gene (a and d), *GmNPR1-1 *(b and e), or *GmNPR1-2 *(c and f) were inoculated with *Pst *just before bolting. a, b, and c were inoculated with a virulent *Pst *strain. d, e, and f, were inoculated with an avirulent *Pst *strain. The plants were infiltrated with either *Pst *DC3000 or *Pst *DC3000 carrying the *AvrRpt2 *gene (10^5 ^cfu/mL (OD_600 _= 0.002). Results were comparable in three independent experiments.

GS_143K5 and GS_101F23 were selected from Class I and Class II BAC clones, respectively. To investigate if *GmNPR1 *genes were orthologous to Arabidopsis *NPR1*, *GmNPR1-1 *and *GmNPR1-2*, isolated from these two BAC clones, were transformed into the Arabidopsis *npr1-1 *mutant carrying the *BGL2-GUS *fusion gene. Transformants were analyzed to confirm the integration of *GmNPR1 *genes into *npr1-1 *by conducting DNA blot analyses. The *npr1-1 *mutant does not induce *PR-1 *transcripts following the SA treatment because it lacks NPR1 function. We investigated if *GmNPR1 *genes, under the control of their native promoters, complemented the *npr1-1 *mutant and mediated the expression of SAR marker gene, *PR-1 *in response to INA treatment. Transgenic Arabidopsis *npr1-1 *mutant plants transformed with either *GmNPR1-1 *or *GmNPR1-2 *showed induction of the Arabidopsis *PR-1 *gene following treatment with INA (Figure 7A). No *PR-1 *transcripts were detected in water controls (Figure 7). These results suggested that *GmNPR1-1 *and *GmNPR1-2 *encode functional NPR1 proteins that were presumably monomerized by INA treatment. The monomeric GmNPR1s then migrated into nuclei and activated transcription of the *PR-1 *gene. In absence of INA, none of the transgenic plants showed any detectible levels of *PR-1 *transcripts. These data suggested that cytosolic GmNPR1 migrated into nucleus following INA treatment [17].

The SAR marker *BGL2 *encoding β-glucanase also requires NPR1 for its induction. The *BGL2-GUS *fusion gene is silent in *npr1-1 *because of the absence of NPR1 function [14]. To determine if *GmNPR1 *genes can complement this lost NPR1 function and initiate pathogen-induced *BGL2 *expression, a transgenic *npr1-1 *mutant plant carrying either *GmNPR1-1 *or *GmNPR1-2 *was tested for expression of GUS driven by the *BGL2 *promoter. Transgenic *npr1-1 *plants carrying either *GmNPR1-1 *or *GmNPR1-2 *were able to show GUS expression when infected with the avirulent *Pst *strain containing *avrRpt2*. These data suggested that both GmNPR1 proteins were able to complement the lost NPR1 function in the *npr1-1 *mutant and induced pathogen-mediated *BGL2 *expression (Figure 7B: e, f). No GUS expression was observed in response to a virulent strain, *Pst *DC3000 carrying no *Avr *genes (Figure 7B: b, c). *BGL2 *expression was observed in the distant healthy tissues of the infected leaves (Figure 7B: e, f). Because of cell death, no GUS expression was detected at the infection sites. Results obtained from three independent experiments strongly suggested that NPR1 function is complemented by both soybean *GmNPR1 *genes in the *npr1-1 *mutant.

**Figure 8:**
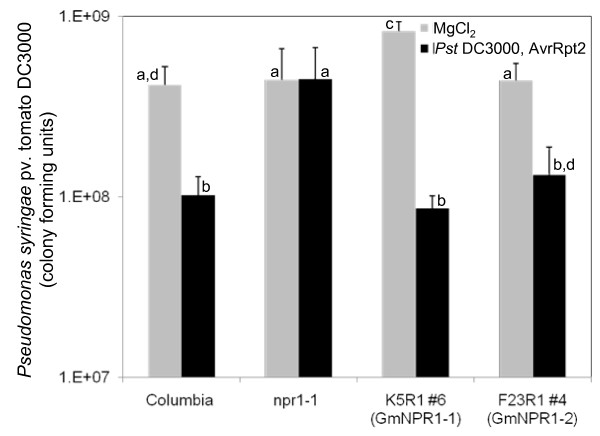
**Induction of SAR in *npr1-1 *plants transformed with *GmNPR1-1 *and *GmNPR1-2 *genes**. Leaf number 3 and 4 were inoculated with 40 μl 10 mM MgCl_2 _or an avirulent strain *Pst DC3000 *containing *AvrRpt2 *(10^7 ^cfu/ml). Three days after inoculation, two younger systemic leaves (leaf number 5 and 6) were inoculated with the virulent strain *Pst *DC3000 (0.5 × 10^5^cfu/ml). Transformants that showed *PR1-1 *expression following INA treatment (e.g. transformant #6 containing *GmNPR1-1 *or transformant #4 containing *GmNPR1-2 *as shown in Figure 7A) also showed SAR activities. The study was conducted with four biological replications. Bars without a common letter on the top are statistically different (Fisher's LSD test, P = 0.05). Standard errors are represented by *error bars*.

To determine if GmNPR1 proteins can induce SAR in non-inoculated leaves, we infected one transformant containing either *GmNPR1-1 *or *GmNPR1-2 *with the bacterial pathogen *Pseudomonas syringae *pv. tomato (*Pst*) DC3000 containing *AvrRpt2*. Three days after inoculation, we inoculated two young non-inoculated leaves with a virulent strain, *Pst *DC3000 and extent of SAR induction in these leaves was determined. Arabidopsis transformants carrying either of the *GmNPR1 *genes showed induction of SAR in response to infection with the avirulent strain, *Pst *DC3000 carrying *AvrRpt2*. There was about 9.5-fold reduction in the number of colony forming units (cfu) of *Pst *in *GmNPR1-1*-complemented plants, when preinoculated with the avirulent strain as compared to the MgCl_2 _control (Figure 8). *GmNPR1-2*, however, resulted in 3.3-fold reduction in numbers of cfu in transformants, preinoculated with the avirulent strain as compared to that in the control (Figure 8). In the avirulent *Pst *strain infected Columbia, GmNPR1-1- and GmNPR1-2-complemented *npr1-1 *plants, significant reduction in cfu of *Pst *was observed when compared to their corresponding MgCl_2 _controls (Figure 8).

## Discussion

### SAR pathway is conserved in soybean

Soybean suffers estimated annual yield loss valued at 2.6 billion dollars from attack of various pathogens [26]. Broad-spectrum SAR has the potentiality to reduce the crop losses from diverse pathogens in soybean. Here we have presented molecular evidence suggesting that the SAR pathway is conserved in soybean. We have isolated soybean genes encoding the SAR regulatory protein, NPR1. Results from Southern blot analysis, gene cloning experiments and soybean genome analyses strongly suggested that there are two *NPR1*-like sequences in soybean. We have also shown that in soybean, SAR marker *GmPR1 *is induced in response to both (i) SAR inducer, INA and (ii) *P. sojae *infection (Figures 1 and 2).

In soybean, SAR activity against *Psg *was induced after two weeks of *P. sojae *infection (Figure 3). However, SAR responses in soybean were not as effective as in some other plant species, such as *Arabidopsis thaliana*, at least in response to the pathogenic infection tested in this investigation [14]. By three weeks following *P. sojae *infection, age-related resistance was expressed in both agar-controls and *P. sojae*-infected seedlings (Figure 3). Age-related resistance has been reported to express in soybean against *P. sojae *[31,32]. Accumulation of SA but not NPR1 is required for this age-related resistance [33].

Soybean is a diploidized tetraploid species. Most likely the two *GmNPR1 *genes were originated from duplication of a single progenitor gene during the polyploidization event. *GmNPR1-1 *and *GmNPR1-2 *with 96% amino acid identity are located in two highly colinear homoeologous chromosomal regions (Additional File 1). RT-PCR data suggested that following duplication, promoter activities of the two genes have been conserved at least for the organs investigated in this study (Figure 6). Both GmNPR1 proteins complemented the lost NPR1 function of the Arabidopsis *npr1-1 *mutant and mediated the expression of *PR-1 *and *BGL2 *following INA treatment and infection, respectively (Figure 7). Further, *GmNPR1*-complemented *npr1-1 *plants were able to show induction of SAR following infection with an avirulent pathogenic strain (Figure 8). From these results we conclude that both *GmNPR1 *genes are orthologous to Arabidopsis *NPR1*.

### Differences in structure-functional regulations of GmNPR1 and Arabidopsis NPR proteins

Arabidopsis NPR1 protein interacts with TGA transcription factors in the nucleus to activate the expression of *PR1 *[34]. Transportation of the NPR1 protein into nucleus is stimulated by SAR inducer [16]. The Arabidopsis *npr1-1 *mutant carrying either the *GmNPR1-1 *or *GmNPR1-2 *showed to initiate *PR-1 *gene expression following treatment with INA (Figure 7). No *PR-1 *induction was observed in the control INA treated mutant *npr1-1 *plant or in the water treated *npr1-1 *plants complemented with either *GmNPR1-1 *or *GmNPR1-2 *(Figure 7). In soybean, INA or infection induced accumulation of *GmPR1 *transcripts (Figures 1 and 2).

In healthy tissues, NPR1 is an oligomeric, cytosolic protein. Following SA treatment, Arabidopsis NPR1 dimers become monomers and move into nuclei to interact with TGA transcription factors for transcriptional activation of *PR1 *[34]. In previous studies it has been shown that Cys^82^, Cys^150^, Cys^155^, Cys^160 ^and Cys^216 ^are involved in oligomer-monomer transition [17,35]. First four of these 5 cysteine residues that are present in BTB/POZ domain of NPR1 are conserved in GmNPR1-1 and GmNPR1-2 (Figure 5). Only Cys^216 ^was not conserved. We used the Cys^216 ^containing region of the *GmNPR1-1 *gene to isolate all available soybean expressed sequence tags and also soybean genome sequence by conducting tBLASTX searches. None of the soybean sequences showed to contain the Arabidopsis Cys^216 ^residue. In this search, we however identified *GmNPR1-1*-*like-1 *and *GmNPR1-1*-*like-2 *genes that are located in two homoeologous chromosomal regions (Additional File 2). Proteins encoded by the two *GmNPR1-1*-*like *genes most unlikely activate the SAR pathway because they are truncated at the N-terminus and do not contain the core BTB/POZ domain required for SA-mediated activation of *PR1 *(Additional File 3; [30]).

In *GmNPR1-1 *and *GmNPR1-2 *transformed *npr1-1 *plants (i) SAR markers *PR1 *and *BGL2 *are induced following INA treatment and infection, respectively and (ii) SAR following infection (Figures 7 and 8). None of the complemented *npr1-1 *mutant plants showed any detectible levels of *PR1 *transcripts prior to INA treatment (Figure 7). These results suggested that GmNPR1 proteins become monomers only following infection or treatment with INA. Thus, either Cys^82^, Cys^150^, Cys^155 ^and Cys^160 ^were sufficient for GmNPR1 oligomerization, or additional cysteine residue(s) may co-operate with Cys^82^, Cys^150^, Cys^155^, and Cys^160 ^for oligomerization of GmNPR1s in soybean or in the *GmNPR1 *complemented *npr1-1 *plants.

In a recent study, S-nitrosylation of Cys^156 ^is shown to play important role in oligomerization of NPR1 in Arabidopsis [35]. In a mutation experiment, where Cys^156 ^was mutated to Asp^156^, the efficiency of oligomer formation was reduced as compared to the wild type protein [35]. In GmNPR1 proteins, although Cys^156 ^was mutated to alanine, both GmNPR1 proteins complemented NPR1 function in the *npr1-1 *mutant (Figure 5). Further investigation is warranted to determine the involvement of other Cystein residues in S-nitrosylation in the absence of Cys^156^.

### Enhancing SAR in soybean

We have shown that SAR marker, *GmPR1 *is expressed in response to both INA treatment and *P. sojae *infection in soybean, and soybean *NPR1 *orthologues are functional. In soybean, it has recently been demonstrated that RAR1 and SGT1 are required for SAR and are functional [36]. Together, these data strongly suggest that SAR is induced in soybean. Therefore, overexpression of *GmNPR1 *genes will most likely enhance broad-spectrum resistance in soybean.

## Conclusion

Complementation analyses in the Arabidopsis *npr1-1 *mutant suggested that homoeologous *GmNPR1-1 *and *GmNPR1-2 *genes are orthologous to Arabidopsis *NPR1*. Therefore, SAR pathway in soybean is most likely regulated by *GmNPR1 *genes. Substitution of essential Cys^216 ^residue for oligomer-monomer transition of Arabidopsis NPR1 with Ser and Leu residues in GmNPR1-1 and GmNPR1-2, respectively suggested that there may be differences between the regulatory mechanisms of GmNPR1 and Arabidopsis NPR proteins. Soybean plants showed expression of the SAR marker *PR1 *gene and SAR following infection, and carry functional *GmNPR1 *genes suggesting that overexpression of *GmNPR1s *in transgenic soybean plants may enhance resistance against many pathogens.

## Methods

### SAR assay following *Phytophthora sojae *infection

The green hypocotyls of 7-day-old light-grown soybean cultivar Williams 82 seedlings were slit open for a length of 1.0 cm and *P. sojae *race 4 mycelia grown in 1/4^th ^strength V8 agar medium were inserted into these wounds [37]. In controls, only agar medium was used to inoculate the wounded hypocotyls. *P. sojae *race 4 is avirulent to Williams 82. Leaves were inoculated with the bacterial pathogen, *Psuedomonas syringae *pv. glycinea (*Psg*), at 9, 13, 17 and 21 days after the inoculation with *P. sojae *race 4 mycellia or agar-with no mycelia. *Psg *cell suspensions (10^7 ^cells/ml) were prepared from 2-day old cell cultures grown in King's B liquid medium [38]. To facilitate bacterial infection, a pricking inoculation technique was used [39]. Ten microliter droplets of either bacterial cell suspensions (10^7 ^cells/ml) or 10 mM magnesium chloride were used to inoculate the youngest trifoliate. Leaves infected with *Psg *were detached 4 days after inoculation. To estimate the size of bacterial population in the inoculated leaves, infected leaves harvested from three different plants per treatment per replication were homogenized in 3 mL 0.9% sodium chloride solution with pestle and mortar. Glycerol stocks were prepared from the homogenized samples and stored at -80°C until use. Different dilutions were plated on King's B medium, grown for 2 days at 27°C and colonies were counted to determine the number of colony forming units in each treatment. Experiment was performed with three biological replications. ANOVA was used to compare different treatments. To determine which of the eight treatments differ from each other, Fisher's least significant difference (LSD) comparisons were performed at P value of 0.05.

### PCR amplification and screening of a soybean BAC library

A soybean EST (Gm-c1004-4231) showing high identity to Arabidopsis *NPR1 *was used to develop a primer pair (forward primer: 5'-GAG CCT TCC ATT ATA GTA TCC CTA CTT AC-3'; reverse primer: 5'-GAC CAG CAA ACT CAG ATG TTG TCT CAG CAT G-3'). The soybean *NPR1*-like sequence, *GmNPR1 *was amplified from Williams 82 genomic DNA by conducting PCR at initial DNA denaturation temperature 94°C for 2 min followed by five cycles of 94°C for 30 sec, 65°C for 30 sec with an increment of -1°C per cycle, 72°C for 1 min; then thirty-five cycles of 94°C for 30 sec, 60°C for 30 sec, 72°C for 1 min, followed by a 10 min DNA extension at 72°C. The amplified products were sequenced to confirm the identity of *GmNPR1 *and used as a probe to screen a soybean Williams 82 BAC library and conduct DNA blot analyses [29].

### DNA gel blot analysis

DNA gel blot analysis was conducted as described previously [40]. DNA was extracted from leaves of the soybean cultivar Williams 82. DNA was digested with four restriction enzymes (*Bcl*I, *Eco*RI, *Hin*dIII, and *Pst*I). Membranes were probed with the ^32^P-radiolabeled *GmNPR1 *sequence [41].

### Cloning *GmNPR1 *genes into the binary vector, pTF101.1

*Eco*RI, *Sst*I, and *Xba*I DNA fragments of two individual BAC clones containing unique *GmNPR1 *sequences were cloned into the binary vector, pTF101.1 in *E. coli *DH10Bα and colonies were screened for DNA fragments containing *GmNPR1 *genes [42]. Resultant plasmids, p143K5Xb1-2.1 and p101F23E1-2 containing *GmNPR1-1 *and *GmNPR1-2 *genes, respectively, under the regulation of their respective native promoters, were selected for further investigation.

### Sequencing of the *GmNPR1-1 *and *GmNPR1-2 *genes

Inserts of p143K5Xb1-2.1 and p101F23E1-2 plasmids containing *GmNPR1-1 *and *GmNPR1-2*, respectively, were sequenced by sub-cloning restriction fragments in the pBluescript II KS (+) vector in *E. coli *DH10Bα. Sequencing was accomplished at the DNA Facility, Iowa State University. Sequence contigs were constructed using ContigExpress™ of the Vector NTI Suite program (InforMax Inc., Bethesda, MD). A primer walking approach was applied in filling the gaps of sequence contigs. *GmNPR1-1*, *GmNPR1-2 *and Arabidopsis *NPR1 *(AAC49611) were compared using ClustalW program (European Bioinformatic Institute). Protein domains were identified by searching the conserved domain database (rpsblast).

### Isolation of soybean *GmNPR1 *cDNAs

A soybean cDNA library was constructed using the pBluescript II XR cDNA library construction kit (Stratagene, La Jolla, CA). Poly(A^+^) RNAs for the cDNA library were prepared from *P. sojae*-infected hypocotyl tissues of Williams 82 by using the polyAtract mRNA isolation system III (Promega, Inc., Madison, WI). The library was constructed in *Eco*RI – *Xho*I sites of the plasmid vector pB42AD (Clontech, Inc., Mountain View, CA). Over 10^6 ^colony forming units (cfu) of the cDNA library were grown on 55 LB agar plates (150 mm × 15 mm) containing ampicillin. cDNAs of the bacterial colonies were blotted onto nylon membranes [42]. Colony blots were hybridized to the radiolabeled *GmNPR1 *probe. Positive colonies were rescreened to identify pure colonies containing single *GmNPR1 *cDNA molecules. Two near full length *GmNPR1 *cDNAs representing both *GmNPR1 *genes were sequenced. Sequences were assembled by ContigExpress™ of the Vector NTI Suite program (InforMax, Inc., Bethesda, MD).

### *GmNPR1 *expressions in soybean organs

Leaf, stem, flower, young pod, and root tissues were collected from Williams 82. Leaf, stem, and root tissues were harvested from three-week old plants. Tissues were frozen quickly in liquid nitrogen and stored at -80°C until their use for RNA isolation. Total RNA was isolated from individual samples using the Qiagen RNeasy Plant Mini kit (Qiagen, Valencia, CA). RNA concentration was determined using a Unico UV-2000 spectrophotometer (Unico, Inc., Dayton, NJ). Gene-specific primers were designed for RT-PCR analyses (*GmNPR1-1*_Forward: GATGCTGACCTTGTTGTCGAGGGAATTC, *GmNPR1-1*_Reverse: CCAGCAAACTCAGATGTTGTCTCAGCATG and *GmNPR1-2_*Forward: GATGCTGACATCGTTGTGGAGGGAATTT, *GmNPR1-2_*Reverse: CCAGCAAAC-TCAGATGTTGTCTCAGCATG). Reverse transcription (RT) was conducted using an oligo-dT primer (TTTTTTTTTTTTTTTTT) and M-MLV reverse transcriptase (Life Technologies, Rockville, MD). A touchdown program used for PCR amplification of *GmNPR1 *was used in RT-PCR analyses. Following five touchdown cycles for primer annealing temperature from 65°C to 60°C, 25 cycles with annealing temperature at 60°C were applied in RT-PCR analyses. PCR products were electrophoresed in 2% agarose gels containing ethidium bromide (0.5 g/mL). The gels were run in 0.5× TBE buffer [42] at 130 volts for 2 h. A 100-bp DNA ladder (Life Technologies, Rockville, MD) was used as a DNA marker. The gels were photographed with an AlphaImager 2000 (Alpha Innotech Corp., San Leandro, CA).

### Transformation of *GmNPR1-1 *and *GmNPR1-2 *into the Arabidopsis *npr1-1 *mutant

Seeds of Arabidopsis *npr1-1 *genotype were obtained from Arabidopsis Biological Resource Center, Ohio State University. Seeds were grown in Sunshine mix SB3000 universal soils (Sun Grow Horticulture Inc., Bellevue, WA) under continuous fluorescent light. Plants were fertilized weekly with the Miracle-Grow Excel water-soluble fertilizer 15-5-15 (Scotts, Marysville, OH). Plasmids p143K5Xb1-2.1 and p101F23E1-2 containing *GmNPR1-1 *and *GmNPR1-2 *genes, respectively, in the binary vector pTF101.1 were transformed into *Agrobacterium tumefaciens *EHA101 by electroporation using a Cell-Porator *Escherichia coli *Pulser (Life Technologies, Rockville, MD). *npr1-1 *mutant was transformed with either p143K5Xb1-2.1 or p101F23E1-2 [43]. Both the genes contained their native promoters.

The T_0 _seedlings were sprayed three times with 200 μM BASTA starting at 15 days after sowing, at a three day interval. The survivors were transferred into new soil. T_1 _seedlings were sprayed three times with 300 μM BASTA at a three day interval starting 21 days following sowing. BASTA resistant plants were used for GUS assays and SAR induction experiments.

### Complementation analysis in transgenic Arabidopsis plants

Ecotype Columbia, *npr1-1 *mutant, and *npr1-1 *mutant transformed with either *GmNPR1-1 *or *GmNPR1-2 *were selected for investigating the complementation of NPR1 function for SAR activity in the *npr1-1 *mutant background. Arabidopsis plants were sown in Sunshine LC1 mix (Sun Grow Horticulture Inc., Bellevue, WA) under 9 h light and 15 h dark regimen at 22°C with 55% humidity. After two weeks, seedlings were transplanted. Four weeks following planting fully developed two leaves (leaf number 3 and 4) were inoculated with 10 mM MgCl_2 _or an avirulent strain *Pst DC3000 *containing *AvrRpt2*. Leaves were inoculated with a syringe containing bacterial cells grown for 48 h in NYG medium containing rifampicin (50 μg/ml) and kanamycin (25 μg/ml) [44]. Bacterial cells for inoculation were collected by centrifugation and then resuspended in 10 mM MgCl_2 _to an optical density 0.2 at A_600_, which corresponds to ~10^8 ^cfu/ml. Bacterial suspensions were diluted to 10^7 ^cfu/ml in 10 mM MgCl_2_[45]. Two leaves per plant were infiltrated with this bacterial suspension using a 1-ml syringe. About 40 μl bacterial suspension (10^7^cfu/ml) was infiltrated in each leaf. Three days after inoculation, two younger systemic leaves (leaf number 5 and 6) were inoculated with the virulent strain *Pst *DC3000. *Pst DC3000 *contains empty vector with the kanamycin resistance gene. Bacterial cells for inoculation were collected by centrifugation and then resuspended in 10 mM MgCl_2 _to an optical density 0.001 at A_600_. After incubation for 3 days at 22°C, the inoculated leaves were harvested. Same size leaf disc was taken from each leaf and was washed twice in sterile water and homogenized in 1 ml 0.9% NaCl. The samples were vortexed and serial dilutions prepared in 0.9% NaCl were plated on NYGA solid medium containing rifampicin (50 μg/ml) and kanamycin (25 μg/ml), and viable colonies were counted after 2 d of growth at 28°C. The study was conducted with four biological replications. Two factor ANOVA was used to compare different treatments. To determine which of the eight treatments differ from each other, Fisher's least significant difference (LSD) comparisons were performed at P value of 0.05.

### Bacterial inoculations and GUS assays of transgenic Arabidopsis plants

*Pst *DC3000 and *Pst *DC3000 carrying the *AvrRpt2 *gene were used for inoculation experiments. The pathogen was grown in NYGA liquid medium containing rifampicin (50 μg/ml) and kanamycin (25 μg/ml) as described above. The leaves of (i) the Arabidopsis *npr1-1 *mutant carrying the *BGL2-GUS *fusion gene or (ii) the *npr1-1 *mutant plants carrying the *BGL2-GUS *and transformed with either *GmNPR1-1 *or *GmNPR1-2 *were infiltrated with either *Pst *DC3000 or *Pst *DC3000 carrying the *AvrRpt2 *gene (10^5 ^cfu/mL (OD_600 _= 0.002). The inoculated leaves were harvested three days after infiltration and stained with X-gluc to localize the GUS activity [46].

### Induction of the *PR-1 *gene transcription in Arabidopsis and soybean

Three-week old Arabidopsis *npr1-1 *(*BGL2-GUS*) mutant or *npr1-1 *(*BGL2-GUS*) transgenic plants containing either *GmNPR1-1 *or *GmNPR1-2 *were uprooted from the soil and washed in water. Roots were dipped in 20 mL ddH_2_O in a 100 × 15 mm Petri dish for 24 h and then water was replaced with 0.5 mM INA for 24 h [17]. Following INA feeding, seedlings were frozen in liquid nitrogen and stored at -80°C until preparation of RNAs. Total RNAs from individual samples were isolated using the Qiagen RNeasy Plant Mini kit (Qiagen, Valencia, CA). RNA concentration was measured using a Unico spectrophotometer (Unico, Dayton, NJ). The same protocol was used for feeding Williams 82 seedlings with INA for various hours in Erlenmeyer flasks.

### Systemic induction of *PR-1 *in soybean

Williams 82 seedlings were grown in trays containing soil. One-week old seedlings were stem-inoculated with the mycelia of *P. sojae *race 4 [47]. Unifoliate and trifoliate leaves, and *P. sojae*-infected tissues were harvested at 0, 1, 2, 3, 4, 5, 6, 9, and 14 days post inoculation, frozen in liquid nitrogen and stored at -80°C until their use for RNA isolation.

### RNA gel blot analysis

Approximately 30 μg total RNAs per sample were fractionated by electrophoresis in 1% formaldehyde-agarose gels and blotted onto Zeta-Probe^® ^GT nylon membranes (Bio-Rad, Hercules, CA) as described earlier [48]. A soybean *PR-1 *gene, *GmPR1 *(AI930866) [12] was used as a probe for the northern blot analyses of soybean RNA samples. The Arabidopsis *PR1 *probe (NM_127025) was PCR amplified from Arabidopsis genomic DNA for northern analyses of Arabidopsis RNA samples. The probes were labeled with α-^32^P (dATP) [41]. Hybridization was carried out at 42°C for 16 to 18 h in buffer used for DNA gel blot hybridization. Membranes were washed twice for five min each in 2× SSC at room temperature followed by three times in washing buffer containing 2× SSC and 0.1% SDS at 65°C for 30 min each before exposure to the X-ray films.

## Authors' contributions

DS carried out SAR experiment in soybean and Arabidopsis, isolated cDNA clones, conducted GUS assays and sequence alignments, participated in designing experiments and drafting the manuscript. IMT carried out DNA and RNA gel blot analyses, screened BAC library, cloned *GmNPR1 *genes into a binary vector, transformed *GmNPR1 *genes into Arabidopsis, conducted sequence alignment and complementation analyses in Arabidopsis and, participated in drafting the initial manuscript and designing experiments. RF participated in SAR experiment in Arabidopsis and GUS assay. MKB participated in designing and coordinating research activities and drafting and finalizing the manuscript. All authors read and approved the final manuscript.

## Supplementary Material

Additional file 1 **Micro-colinearity between homoeologous regions containing *GmNPR1*-like sequences**. mVISTA (; [49]) program was used to determine the micro-colinearity between Scaffold_159 and Scaffold-213 carrying *GmNPR1-1 *and *GmNPR1-2*, respectively. The location of the *GmNPR1 *sequences is shown with a black box. The extent of identity between conserved sequences at the *GmNPR1 *region is around 70%. Click here for file

Additional file 2 **Micro-colinearity between homoeologous regions containing *GmNPR1-1-like *sequences**. mVISTA (; [49]) program was used to determine the micro-colinearity between Scaffold_15 and Scaffold-90 carrying *GmNPR1-1-like-1 *and *GmNPR1-1-like-2*, respectively. The location of the *GmNPR1-1-like *sequences is shown with a black box between 32 and 34 kb sequence of Scaffold_15, which was the sequence 1 in the mVISTA analysis. The extent of identity between conserved genic sequences is around 70%. Click here for file

Additional file 3 **Comparison of Soybean *NPR1*-like sequences with Arabidopsis NPR1**. *GmNPR1-1*-like sequence (BE801977.1) was used to identify two GmNPR1-like peptides, GmNPR1-like-1 (Gm0015x00979.1:peptide; ) and GmNPR1-like-2 (Gm0090x00318:peptide; ) from Scaffold_15 and Scaffold_90 of the soybean genome sequence, respectively (). ClustalW analysis (; [50]) revealed that these two peptides along with GmNPR1-1, GmNPR1-2, NPR3 (NP_199324.2), NPR4 (NP_193701.2) do contain the Cys^216 ^residue (red font) essential for oligomerization of NPR1 (NP_176610.1). Click here for file
