# Supplementary figures and images for: Systemic acquired resistance in soybean is regulated by two proteins, Orthologous to Arabidopsis NPR1
Source: BMC Plant Biol. 2009 Aug 5;9:105. doi: 10.1186/1471-2229-9-105 (PMC2738679; doi:10.1186/1471-2229-9-105)

## Slide 1
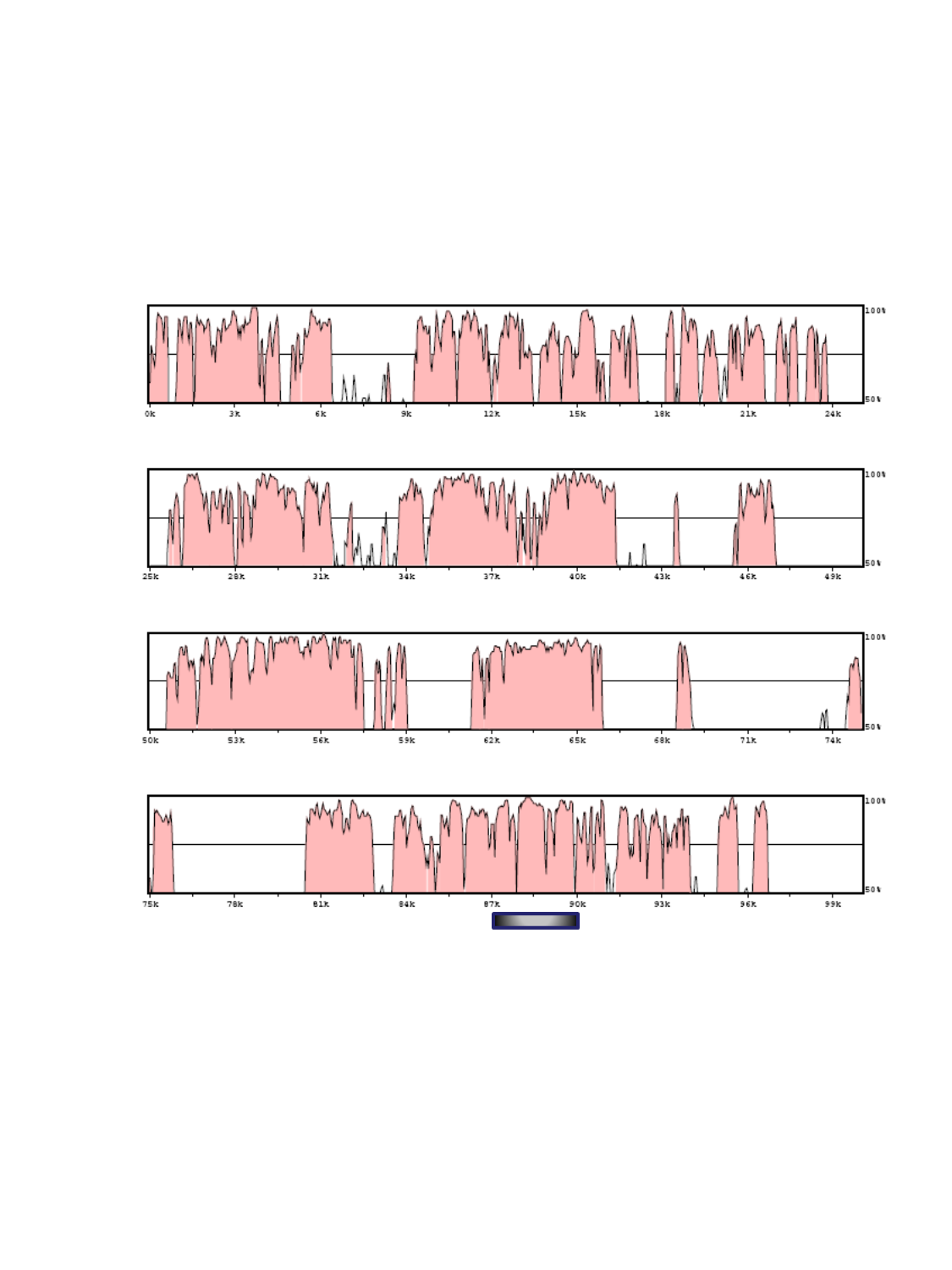

Supplement: Additional file 1 — Micro-colinearity between homoeologous regions containing GmNPR1-like sequences. mVISTA (; [49]) program was used to determine the micro-colinearity between Scaffold_159 and Scaffold-213 carrying GmNPR1-1 and GmNPR1-2, respectively. The location of the GmNPR1 sequences is shown with a black box. The extent of identity between conserved sequences at the GmNPR1 region is around 70%. [file 1471-2229-9-105-S1.ppt]

## Slide 1
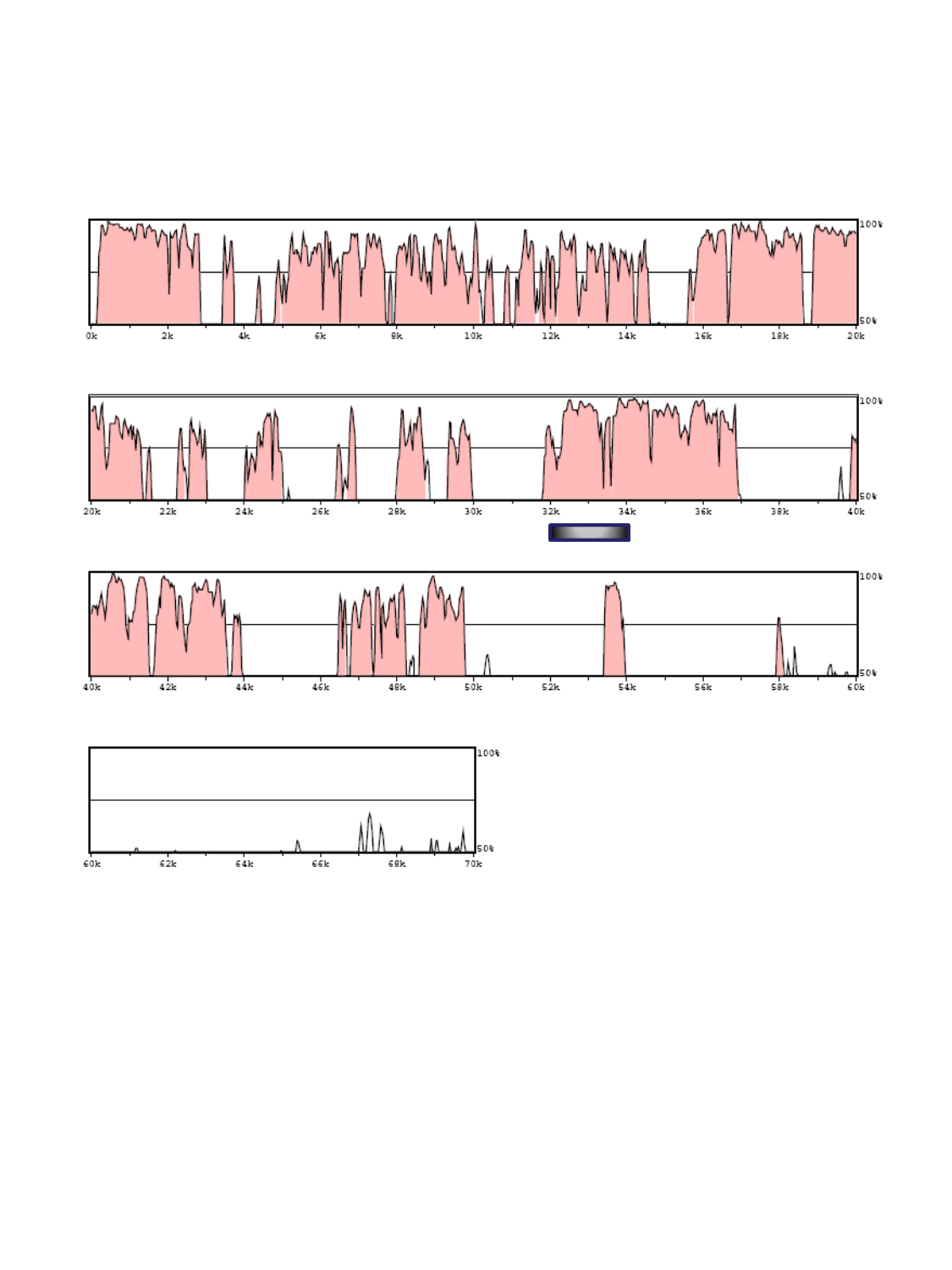

Supplement: Additional file 2 — Micro-colinearity between homoeologous regions containing GmNPR1-1-like sequences. mVISTA (; [49]) program was used to determine the micro-colinearity between Scaffold_15 and Scaffold-90 carrying GmNPR1-1-like-1 and GmNPR1-1-like-2, respectively. The location of the GmNPR1-1-like sequences is shown with a black box between 32 and 34 kb sequence of Scaffold_15, which was the sequence 1 in the mVISTA analysis. The extent of identity between conserved genic sequences is around 70%. [file 1471-2229-9-105-S2.ppt]

## Slide 1
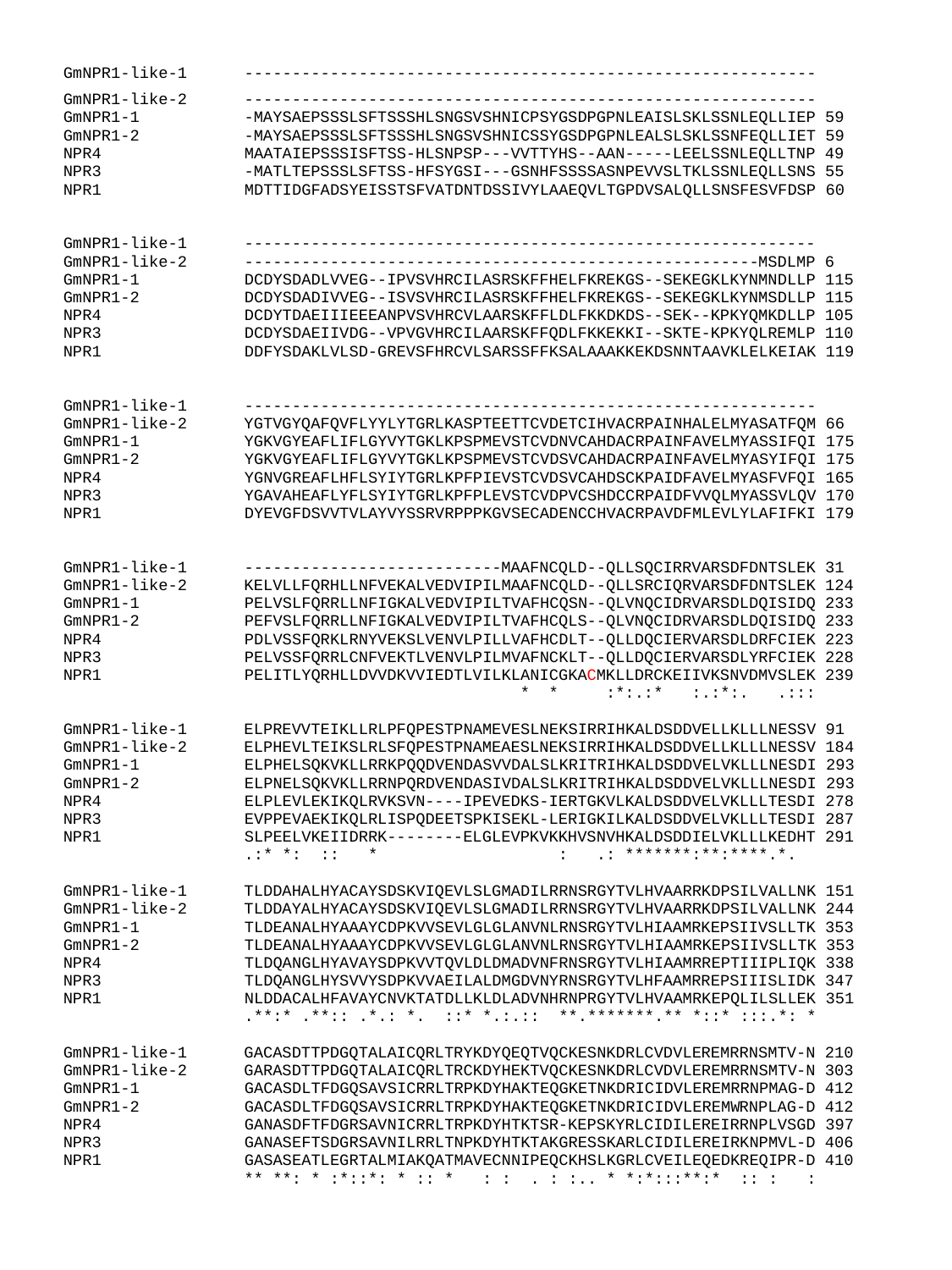

## Slide 2
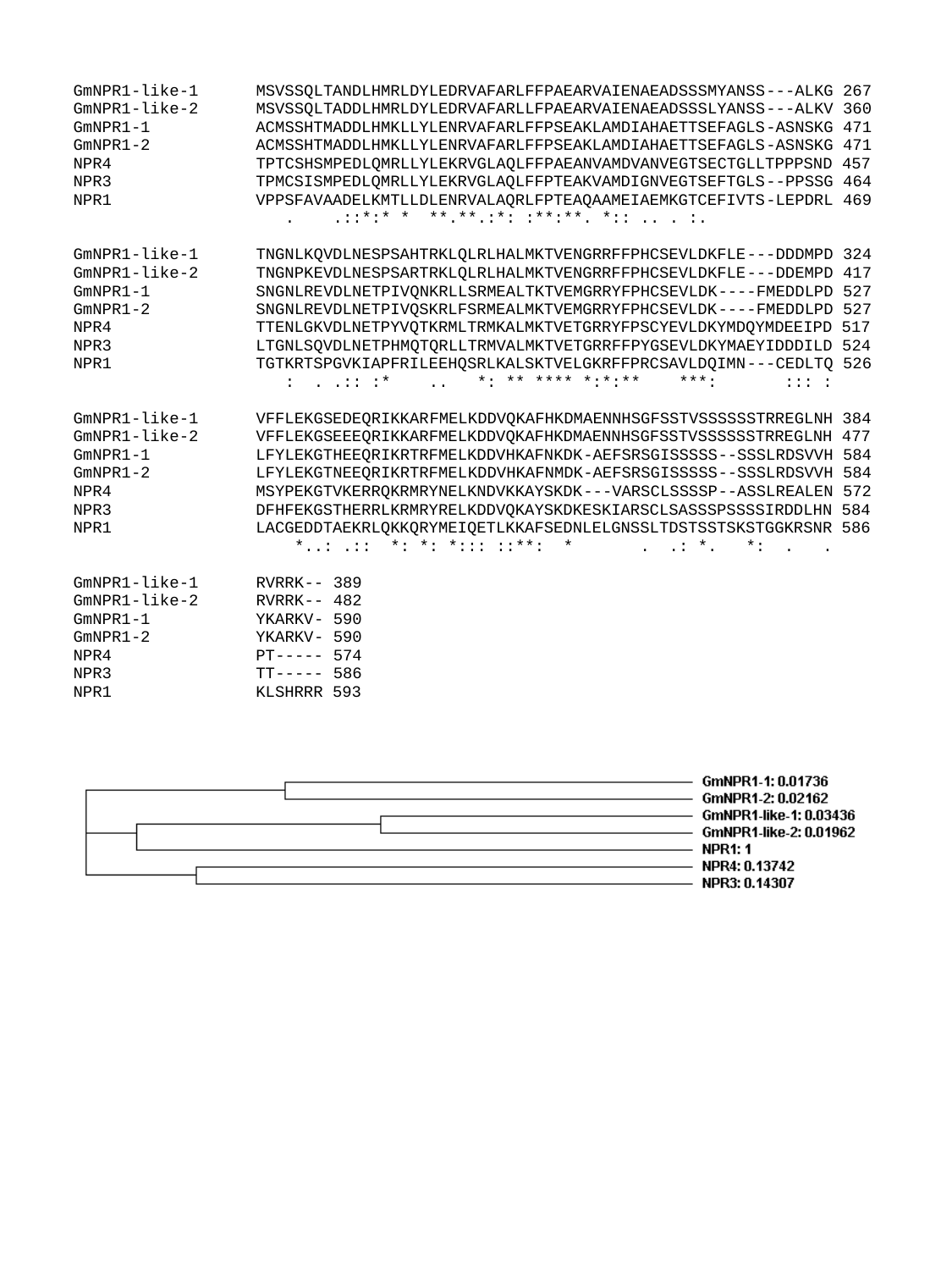

Supplement: Additional file 3 — Comparison of Soybean NPR1-like sequences with Arabidopsis NPR1. GmNPR1-1-like sequence (BE801977.1) was used to identify two GmNPR1-like peptides, GmNPR1-like-1 (Gm0015x00979.1:peptide; ) and GmNPR1-like-2 (Gm0090x00318:peptide; ) from Scaffold_15 and Scaffold_90 of the soybean genome sequence, respectively (). ClustalW analysis (; [50]) revealed that these two peptides along with GmNPR1-1, GmNPR1-2, NPR3 (NP_199324.2), NPR4 (NP_193701.2) do contain the Cys216 residue (red font) essential for oligomerization of NPR1 (NP_176610.1). [file 1471-2229-9-105-S3.ppt]
